# Identification of the Characteristic Genes and their Roles in Lung Adenocarcinoma Lymph Node Metastasis through Machine Learning Algorithm

**DOI:** 10.1155/2022/1968829

**Published:** 2022-10-12

**Authors:** Qian Zhou, Xianghui Wang, Haiyun Qian, Shengwei Ma, Chenggang Lei, Fenghe Cui

**Affiliations:** Department of Cardiothoracic Surgery, Jingzhou Central Hospital, Jingzhou Hospital Affiliated to Yangtze University, Jingzhou, Hubei, China

## Abstract

**Background:**

Lymph node metastasis is an important route of lung cancer metastasis and can significantly affect the survival of lung cancer.

**Methods:**

All the analysis was conducted out in the R software. Expression profile and clinical information of lung adenocarcinoma (LUAD) patients were downloaded from The Cancer Genome Atlas database.

**Results:**

In our study, we firstly identified the characteristic genes of lymph node metastasis in LUAD through two machine learning algorithms, least absolute shrinkage and selection operator (LASSO) logistic regression, and SVM-RFE algorithms. Ten characteristic genes were finally identified, including *CRHR2*, *ITIH1*, *PRSS48*, *MAS1L*, *CYP4Z1*, *LMO1*, *TCP10L2*, *KRT78*, *IGFBP1*, and *PITX3*. Next, we performed univariate Cox regression, LASSO regression, and multivariate Cox regression sequentially to construct a prognosis model based on *MAS1L*, *TCP10L2*, and *CRHR2*, which had a good prognosis prediction efficiency in both training and validation cohorts. Univariate and multivariate analysis indicated that our model is a risk factor independent of other clinical features. Pathway enrichment analysis showed that in the high-risk patients, the pathway of MYC target, unfolded protein response, interferon alpha response, DNA repair, reactive oxygen species pathway, and glycolysis were significantly enriched. Among three model genes, *MAS1L* aroused our interest and therefore was selected for further analysis. KM survival curves showed that the patients with higher *MAS1L* might have better disease-free survival and progression-free survival. Further, pathway enrichment, genomic instability, immune infiltration, and drug sensitivity analysis were performed to in-deep explore the role of *MAS1L* in LUAD.

**Conclusions:**

Results showed that the signature based on *MAS1L*, *TCP10L2*, and *CRHR2* is a useful tool to predict prognosis and lung cancer lymph node metastasis.

## 1. Introduction

Lung cancer is a leading cause of cancer-related deaths all over the world, in which non-small-cell lung cancer (NSCLC) is the most common pathological subtype and accounts for approximately 85% of cases [1]. Many factors may contribute to the occurrence and development of lung cancer, including smoking, genetic susceptibility, environmental exposure, and so on [2]. Surgery can significantly improve the prognosis of early-stage and resectable lung cancer patients. Unfortunately, inadequate screening plans and hidden clinical symptoms have resulted in most patients being diagnosed with advanced disease at the time of their first presentation [3]. However, for those patients in the advanced stage, the prognosis is still unsatisfactory [3]. Therefore, it is meaningful to identify novel molecules associated with patient's prognosis and with the potential to guide therapy options.

Lymph node metastasis is an important feature of lung cancer and is associated with poor prognosis [4]. In the absence of distant metastasis, an accurate assessment of lymph node involvement is a crucial step for NSCLC patients [4]. Throughout the last decades, radical lobectomy has been universally accepted as a standard procedure for lung cancer patients [5]. In recent years, intrathoracic reassessments during thoracotomies for lung cancer have evolved into detailed and complex assessments, and the core of which is to evaluate the involvement of lymph nodes at the mediastinal and hilar levels [5]. This technique is called “systematic lymph node dissection”, which has been accepted as an important part of lung cancer surgery. At the genome level, researchers have focused on the molecules involved in lung cancer lymph node metastasis that might be the underlying therapy target. For instance, Liu et al. found that in small cell lung cancer, patients with high *CCL19* expression had poorer outcomes and more LN metastasis, as well as impaired CD8+ T cell function [6]. Moreover, Bi et al. revealed that *CXCR4* and *VEGF-C* are correlated with lymph node metastasis and might synergistically promote lung cancer progression [7]. Thus, exploring the potential mechanism of lymph node metastasis of lung cancer from a molecular perspective is helpful for the diagnosis and treatment of lung cancer.

In our study, we firstly identified the characteristic genes of lymph node metastasis in LUAD through least absolute shrinkage and selection operator (LASSO) logistic regression and SVM-RFE algorithms. A prognosis model based on *MAS1L*, *TCP10L2*, and *CRHR2* was then established, which had a good prognosis prediction efficiency in both training and validation cohorts. Next, pathway enrichment analysis was performed to explore the underlying biological differences between high- and low-risk patients. Among three model genes, *MAS1L* aroused our interest and therefore was selected for further analysis. KM survival curves showed that the patients with higher *MAS1L* might have better disease-free survival and progression-free survival. Further, pathway enrichment, genomic instability, immune infiltration, and drug sensitivity analysis were performed to in-deep explore the role of *MAS1L* in LUAD.

## 2. Methods

### 2.1. Data Acquisition

The open-accessed transcriptional profiling data and clinical information of lung adenocarcinoma (LUAD) were downloaded from The Cancer Genome Atlas (TCGA, https://portal.gdc.cancer.gov/; 522 patients, age: 65.33 ± 10.02) database. The original expression profile files were “STAR-counts” form and further sorted using the author's R code (tpm_unstranded). Clinical information was collated using Perl code. Genomic reference file GRCh38.gtf was used for probe annotation. The baseline information of patients was shown in Table 1. For the enrolled patients, the patients with N0 stage was regarded as the population without lymph node metastasis, while the N1-3 stage patients were regarded as the population with lymph node metastasis.

### 2.2. Machine Learning Algorithm

LASSO logistic regression and support vector machine recursive feature elimination (SVM-RFE) algorithms were used for characteristic genes screening. LASSO logistic regression was performed based on the glmnet package [8]. SVM is a machine learning method that can find the best variables by deleting the feature vectors generated by SVM [9].

### 2.3. Establishment of Prognosis Model

Patients were randomly assigned to training and validation cohorts with a 1 : 1 ratio. Based on the characteristic genes identified by LASSO logistic regression and SVM-RFE algorithms, univariate Cox regression analysis was firstly performed to determine prognosis-related genes with the threshold of *P* < 0.05. Next, LASSO regression was used for dimensionality reduction [10]. Finally, multivariate Cox regression analysis was utilized for prognosis model construction with the formula of “Riskscore = Gene A^∗^Coef A + Gene B ^∗^ Coef B + … + Gene N ^∗^ Coef N”. Each patient would be assigned a riskscore according to the above formula [11].

### 2.4. Model Evaluation

According to the calculated riskscore, the patients with riskscore higher than the median were defined as high-risk group, otherwise, the low-risk group. Kaplan-Meier (KM) survival curve was used to evaluate the prognosis difference between high- and low-risk patients. The receiver operating characteristic (ROC) curve was used to evaluate the prediction efficacy of our model in a specific time node. The independence of our prognosis model was validated by univariate and multivariate Cox regression analysis [12].

### 2.5. Pathway Enrichment and Genomic Instability Analysis

Underlying biological differences can lead to different outcomes in patients. Pathway enrichment analysis was performed based on the GSEA algorithm. The reference gene set was set as Hallmark, c2.cp.kegg.v7.5.1.symbols, and c5.go.v7.5.1.symbols gene set. ClueGO plug-in in cytoscape software was used for the gene pathways enrichment [13]. The data of tumor mutational burden (TMB) and microsatellite instability (MSI) were downloaded from the TCGA database. The tumor stemness index (mRNAsi and EREG-mRNAsi) of TCGA-LUAD patients was obtained from the previous study [14].

### 2.6. Immune-Related and Drug Sensitivity Analysis

Immune infiltration analysis was conducted using the single sample gene set enrichment analysis (ssGSEA) algorithm [15]. Evaluation of the sensitity on immunotherapy was performed using the tumor immune dysfunction and exclusion (TIDE) analysis, in which the TIDE score < 0 was defined as the immunotherapy responders, and > 0 was regarded as the nonresponders [16]. Drug sensitivity analysis was performed based on the Genomics of Drug Sensitivity in Cancer (GDSC) database [17].

### 2.7. Statistical Analysis

All the analysis was performed using the R software version 4.0.0. Values of *P* < 0.05 were considered statistically significant. Normal distribution was tested by the Student *t*-test. Nonnormally distributed variables were compared using the Mann–Whitney *U* test.

## 3. Result

### 3.1. Identification of the Characteristic Genes of Lymph Node Metastasis

**Figure 1:**
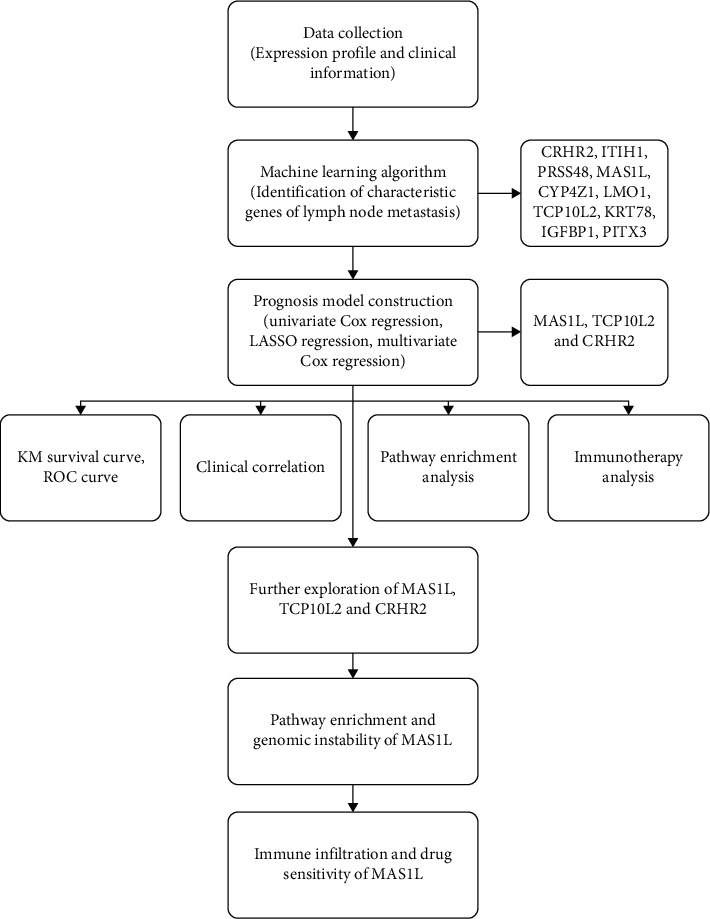
The flow chart of the whole study.

**Figure 2:**
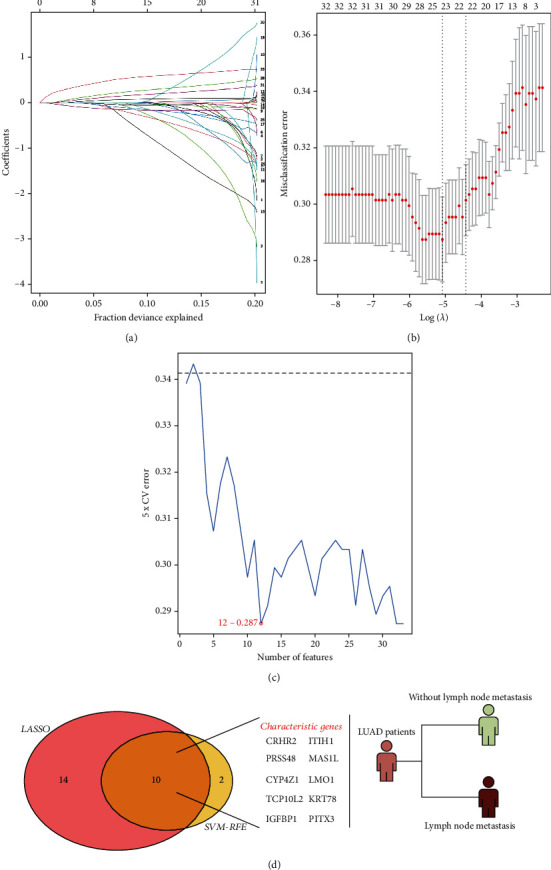
Identification of the characteristic genes of lung cancer lymph node metastasis. Notes: (a–b) LASSO logistic regression; (c) SVM-RFE algorithm; (d) ten characteristic genes were identified based on LASSO logistic regression and SVM-RFE algorithms, including *CRHR2*, *ITIH1*, *PRSS48*, *MAS1L*, *CYP4Z1*, *LMO1*, *TCP10L2*, *KRT78*, *IGFBP1*, and *PITX3.*

The flow chart of the whole study was shown in Figure 1. For the obtained data of TCGA-LUAD patients, we divided then into lymph node metastasis (N1-3) and non-lymph node metastasis (N0) group. LASSO logistic regression and SVM-RFE algorithms were used to identify the characteristic gene of lymph node metastasis (Figures 2(a–c)). Finally, LASSO logistic regression and SVM-RFE algorithms intersected ten characteristic genes, including *CRHR2*, *ITIH1*, *PRSS48*, *MAS1L*, *CYP4Z1*, *LMO1*, *TCP10L2*, *KRT78*, *IGFBP1*, and *PITX3* (Figure 2(d)).

### 3.2. Prognosis Model Construction

**Figure 3:**
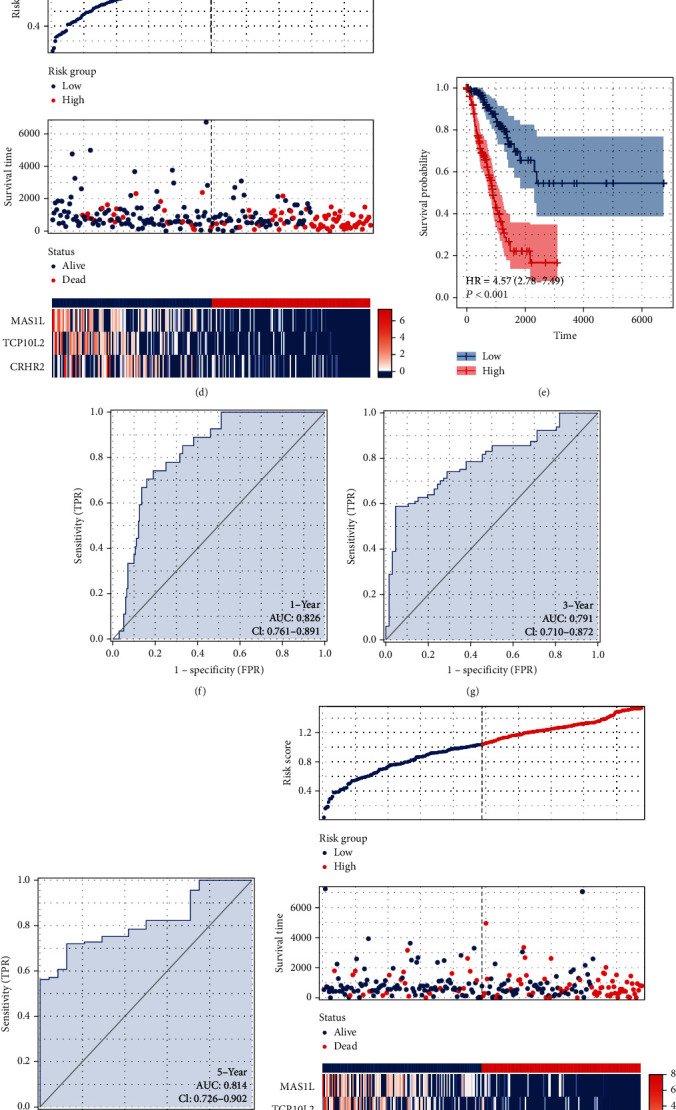
Prognosis model construction. Notes: (a) univariate Cox regression analysis of the identified characteristic genes to select prognosis-related genes; (b) LASSO regression was used for dimensionality reduction; (c) multivariate Cox regression analysis identified three genes *MAS1L*, *TCP10L2*, and *CRHR2* for model construction; (d) overview of the riskscore in the training model; (e) KM survival curve of high- and low-risk patients in training cohort; (f–h) ROC curves of 1-, 3-, and 5-years survival in the training cohort; (i) overview of the riskscore in the validation model; (j) KM survival curve of high- and low-risk patients in validation cohort; (k–m) ROC curves of 1-, 3-, and 5-years survival in the validation cohort.

Next, univariate Cox regression analysis was performed to identify the prognosis-related characteristic genes. The result showed that among all these ten genes, *IGFBP1*, *TCP10L2*, *MAS1L*, *CYP4Z1*, and *CRHR2* were the protective factors, while *PITX3* was the risk factor (Figure 3(a)). LASSO regression was then used for data dimensionality reduction (Figure 3(b)). Multivariate Cox regression analysis identified three genes for prognosis model construction, including *MAS1L*, *TCP10L2*, and *CRHR2* (Figure 3(c)). In the training cohort, a higher proportion of dead cases were observed in the high-risk group (Figure 3(d)). KM survival curve showed that the high-risk patient might have a poor prognosis compared to the patients in low-risk group (Figure 3(e)). ROC curves showed a great prediction efficiency of patients 1-, 3-, and 5-year survival (Figures 3(f–h), 1-year AUC = 0.826, 3-year AUC = 0.791, and 5-year AUC = 0.814). Meanwhile, the same trend was also found in the validation group (Figure 3(i)). KM survival curve showed that in the validation group, the high-risk patient might have a wose prognosis (Figure 3(j)). Also, ROC curves showed a good prediction efficiency of patients' 1-, 3-, and 5-year survival in validation group (Figures 3(k–m), 1-year AUC = 0.672, 3-year AUC = 0.751, and 5-year AUC = 0.7).

### 3.3. Clinical Correlation

**Figure 4:**
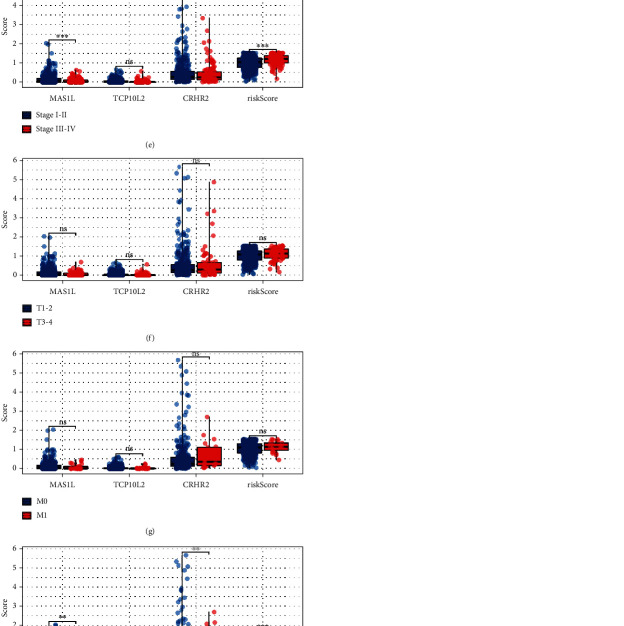
Clinical correlation of our model. Notes: (a–b) univariate and multivariate analysis showed that the model is a risk factor independent of other clinical features; (c) the expression level of *MAS1L*, *TCP10L2*, *CRHR2*, and riskscore in patients with different age groups, ns = *P* > 0.05; (d) The expression level of *MAS1L*, *TCP10L2*, *CRHR2*, and riskscore in patients with different gender groups, ns = *P* > 0.05, ^∗^ = *P* < 0.05, and ^∗∗^ = *P* < 0.01; (e) the expression level of *MAS1L*, *TCP10L2*, *CRHR2*, and riskscore in patients with different stage groups, ns = *P* > 0.05 and ∗∗∗ = *P* < 0.001; (f) the expression level of *MAS1L*, *TCP10L2*, *CRHR2*, and riskscore in patients with different T-stage groups, ns = *P* > 0.05; (g) the expression level of *MAS1L*, *TCP10L2*, *CRHR2*, and riskscore in patients with different M-stage groups, ns = *P* > 0.05; (h) the expression level of *MAS1L*, *TCP10L2*, *CRHR2*, and riskscore in patients with different N-stage groups, ^∗^ = *P* < 0.05, ^∗∗^ = *P* < 0.01, and ^∗∗∗^ = *P* < 0.001.

Univariate and multivariate analysis showed that our model is a risk factor independent of other clinical features (Figures 4(a) and 4(b)). Then, we performed the clinical correlation of our model, as well as the model genes. No significant difference was found in model genes and riskscore between <= 65 and > 65 patients (Figure 4(c)); MAS1L was higher expressed in female patients compared with the male patients (Figure 4(d)); MAS1L was higher expressed in stage I-II patients compared with the stage III-IV patients (Figure 4(e)); no significant difference was found in model genes and riskscore between different T-stage patients (Figure 4(f)); no significant difference was found in model genes and riskscore between different M-stage patients (Figure 4(g)); N1-3 patients had a lower *MAS1L*, *TCP10L2*, and *CRHR2* expression, but a higher riskscore (Figure 4(h)).

### 3.4. Pathway Enrichment Analysis and Immunotherapy Analysis

**Figure 5:**
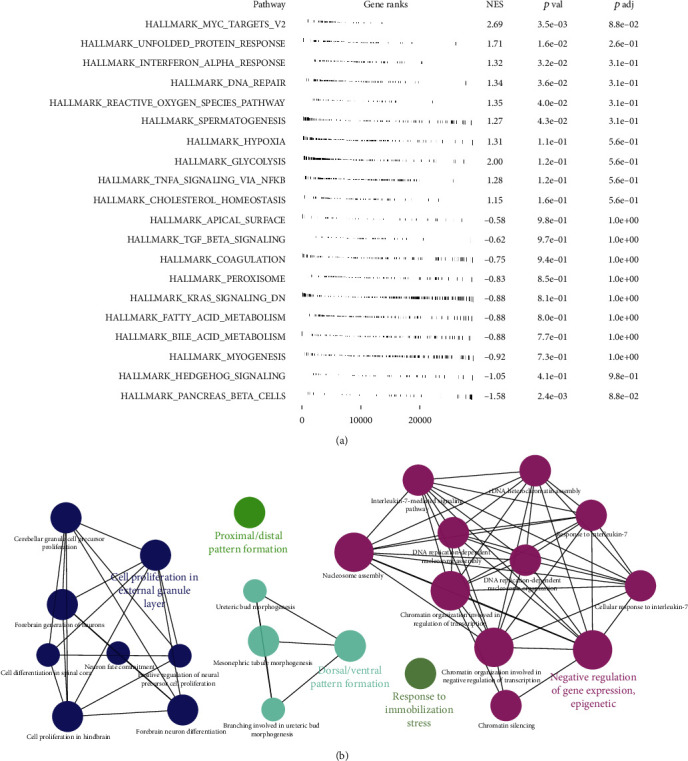
Pathway enrichment analysis. Notes: (a) GSEA analysis of high-risk based on the Hallmark gene set; (b) ClueGO analysis of the high-risk.

We next explored the underlying biological differences between high- and low-risk patients. GSEA analysis showed that in the high-risk patients, the pathway of *MYC* target, unfolded protein response, interferon alpha response, DNA repair, reactive oxygen species pathway, and glycolysis were significantly enriched in. (Figure 5(a)). ClueGO analysis showed that our model was mainly enriched in cell proliferation in external granule layer, proximal/distal pattern formation, dorsal/ventral pattern formation, response to immobilization stress, and negative regulation of gene expression and epigenetic (Figure 5(b)). Gene ontology (GO) analysis showed that in the high-risk patients, the terms of DNA replication checkpoint signaling, DNA strand elongation involved in DNA replication, positive regulation of telomerase RNA localization to cajal body, DNA replication origin binding, anaphase promoting complex dependent catabolic process, establishment of protein localization to telomere, and kinetochore assembly were significantly enriched (Figure S2A). Kyoto Encyclopedia of Genes and Genomes (KEGG) analysis indicated that mismatch repair, citrate cycle TCA cycle, homologous recombination, DNA replication, proteasome, and ribosome were significantly enriched (Figure S2B). A positive correlation was found between TIDE and riskscore (Figure S1A, Correlation = 0.193, *P* < 0.001). Meanwhile, a higher TIDE score was found in the high-risk patients, indicating a lower percentage of immunotherapy responders in high-risk group (Figure S1B-C, 23.1% vs. 43.4%).

### 3.5. Further Exploration of *MAS1L*, *TCP10L2*, and *CRHR2*

**Figure 6:**
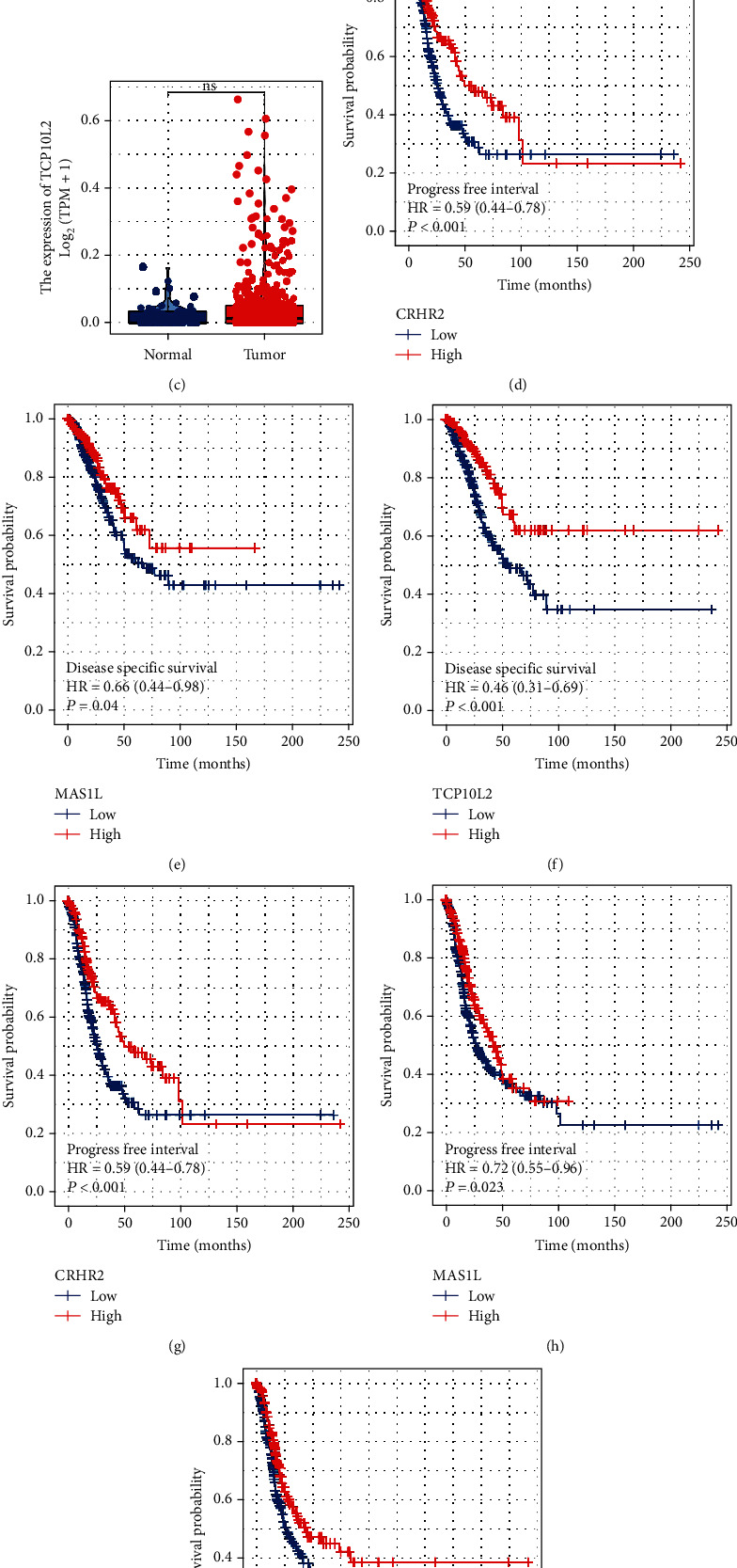
Further exploration of *MAS1L*, *TCP10L2*, and *CRHR2*. Notes: (a–c) the expression level of *MAS1L*, *TCP10L2*, and *CRHR2* in normal and tumor tissue; (d–f) disease-specific survival of *MAS1L*, *TCP10L2*, and *CRHR2*; (g–i) progression-free survival of *MAS1L*, *TCP10L2*, and *CRHR2*.

**Figure 7:**
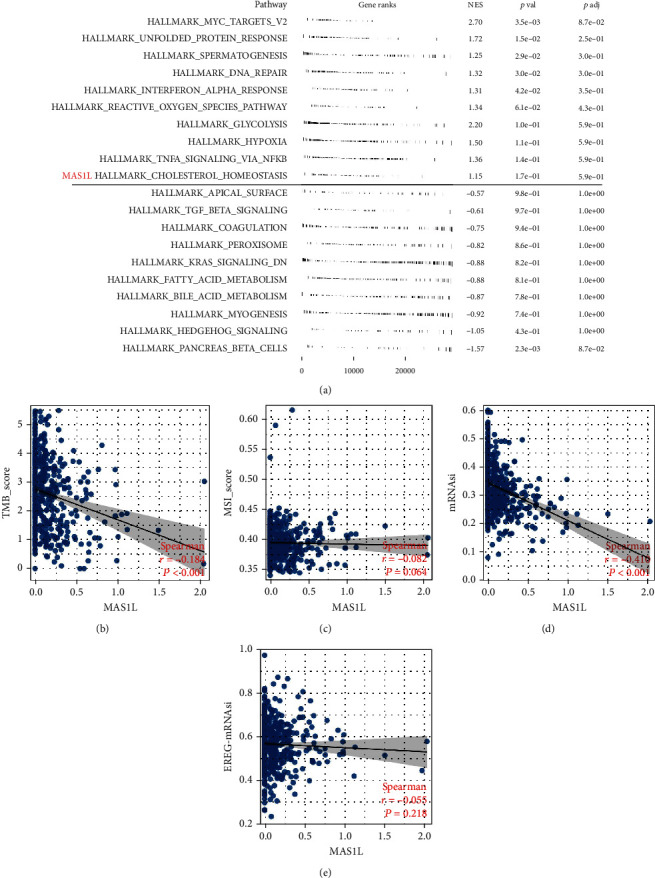
Pathway enrichment and genomic instability of MAS1L. Notes: (a) GSEA analysis of high and low MAS1L based on the Hallmark gene set; (b–e) the correlation between riskscore and TMB, MSI, mRNAsi, and EREG-mRNAsi.

Furthermore, we tried to compare the expression level of *MAS1L*, *TCP10L2*, and *CRHR2* in normal and LUAD samples (Figures 6(a–c)). The result showed that *MAS1L* was significantly downregulated in LUAD samples (Figure 6(b)). Moreover, KM survival curves showed that the patients with higher *MAS1L, TCP10L2*, and *CRHR2* might have better disease-free survival and progression-free survival (Figures 6(d–i)). *MAS1L* aroused our interest and therefore selected for further analysis. Pathway enrichment analysis showed that in the patients with high MAS1L expression, the pathway of apical surface, TGF-*β* signaling, coagulation, peroxisome, KRAS signaling, fatty acid metabolism, and bile acid metabolism hedgehog signaling were downregulated (Figure 7(a)). GO analysis showed that the terms of RNA binding involved in posttranscriptional gene silencing, T cell receptor complex, plasma membrane signaling receptor complex, bitter taste receptor activity, spliceosomal tri snrnp complex assembly, and cajal body were significantly enriched in patients with high *MAS1L* level (Figure S3A). KEGG analysis showed that the terms of intestinal immune network for iga production, asthma, allograft rejection, hematopoietic cell lineage, viral myocarditis, and autoimmune thyroid disease were significantly enriched in the patients with high *MAS*1*L* level (Figure S3B). Next, we explored the correlation between *MAS*1*L* and genomic instability (Figures 7(b–e)). A negative correlation was found between *MAS*1*L* and TMB score and mRNAsi (Figures 7(b) and 7(r) = -0.184, *P* < 0.001; Figures 7(d) and 7(r) = -0.416, *P* < 0.001). However, no remarkable correlation was observed between the *MAS1L* and MSI score and EREG-mRNAsi (Figures 7(c) and 7(e)).

### 3.6. Immune Infiltration and Drug Sensitivity of *MAS1L*

**Figure 8:**
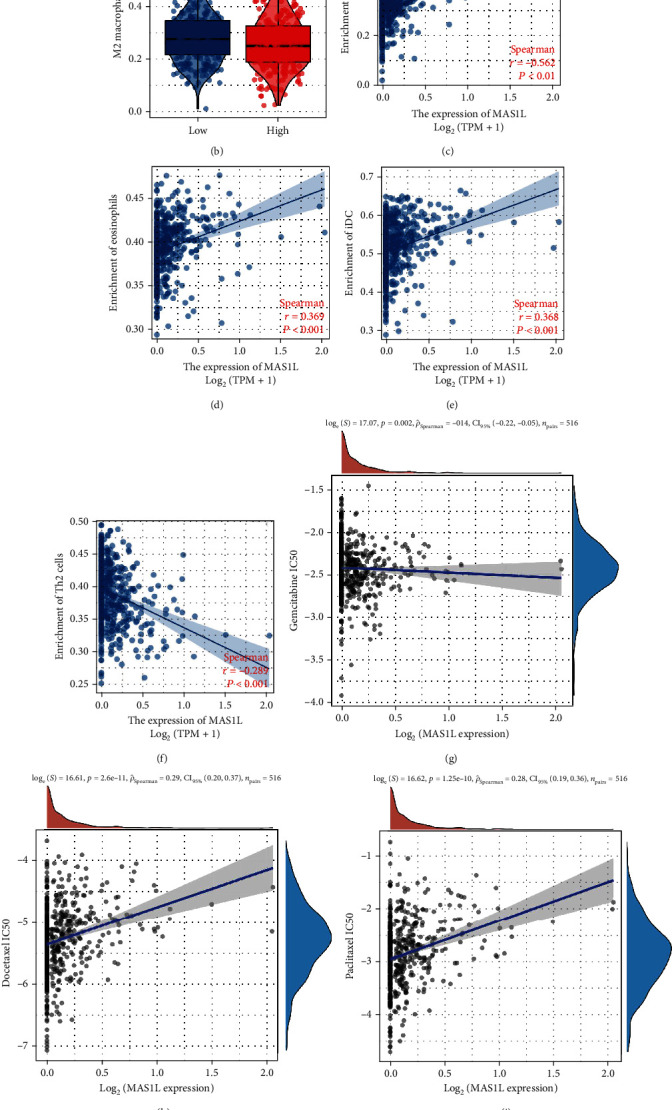
Immune infiltration and drug sensitivity analysis of *MAS1L*. Notes: (a) the CIBERSORT algorithm was used to quantify the immune infiltration of cancer tissue; (b) the patients with high *MAS1L* might have a lower M2 macrophages infiltration; (c–f) the correlation of riskscore and mast cells, eosinophil, iDC, and Th2 cells; (g–j) the correlation of riskscore and the IC50 of Gemcitabine, Docetaxel, Paclitaxel, and Cisplatin.

Immune microenvironment played an important role in tumor development. Immune infiltration analysis showed that riskscore was positively correlated with the mast cells, eosinophils, iDC, DC, and macrophages, while negatively correlated with Th2 cells (Figures 8(a) and 8(c–f)). Also, we found that the patients with high *MAS1L* expression might have a higher M2 macrophages infiltration (Figure 8(b)). Moreover, we performed drug sensitivity analysis to explore the underlying effect of MAS1L on the chemotherapeutic drugs of lung cancer (Figures 8(g–j)). The result indicated that the patients with high *MAS1L* expression might have a lower sensitivity to docetaxel and paclitaxel (Figures 8(h) and 8(i)).

## 4. Discussion

Lung cancer is a serious public health concern worldwide [1]. Lymph node metastasis is common in lung cancer and regarded as an independent prognosis factor [18]. However, the underlying biological mechanisms affecting the lymph node metastasis of lung cancer have not been fully explored.

In our study, we firstly identified the characteristic genes of lymph node metastasis in LUAD through two machine learning algorithms, LASSO logistic regression and SVM-RFE algorithms. Ten characteristic genes were finally identified, including *CRHR2*, *ITIH1*, *PRSS48*, *MAS1L*, *CYP4Z1*, *LMO1*, *TCP10L2*, *KRT78*, *IGFBP1*, and *PITX3*. In the clinical practice, detecting the relative expression of these genes can indicate the risk group of patients, as well as their response to chemotherapy and immunotherapy.

Next, we performed univariate Cox regression, LASSO regression, and multivariate Cox regression sequentially to construct a prognosis model based on *MAS1L*, *TCP10L2*, and *CRHR2*, which had a good prognosis prediction efficiency in both training and validation cohorts. Univariate and multivariate analysis indicated that our model is a risk factor independent of other clinical features. Next, pathway enrichment analysis was performed to explore the underlying biological differences between high- and low-risk patients. Among three model genes, *MAS1L* aroused our interest and therefore was selected for further analysis. KM survival curves showed that the patients with higher MAS1L might have better disease-free survival and progression-free survival. Further, pathway enrichment, genomic instability, immune infiltration, and drug sensitivity analysis were performed to in-deep explore the role of *MAS1L* in LUAD.

Our result showed that the pathway of MYC target, unfolded protein response, interferon alpha response, DNA repair, reactive oxygen species pathway, and glycolysis were significantly enriched in the high-risk patients. A break in the balance of DNA damage and repair would lead to the accumulation of oncogenes in tumor cells, leading to genomic instability and malignant progression [19]. In lung cancer, Tian et al. found that targeting UHRF1-dependent DNA repair could selectively sensitize *KRAS* mutant lung cancer to chemotherapy [20]. Glycolysis is widely involved in the development of lung cancer. Hua et al. revealed that LINC01123 could facilitate growth and aerobic glycolysis of lung cancer through the miR-199a-5p/c-Myc axis [21]. Wiel et al. found that *BACH1* can activate the transcription of hexokinase 2 and *GAPDH* and increases glucose uptake, glycolysis rate, and lactate secretion, thereby stimulating glycolysis-dependent metastasis of lung cancer cells [22]. Zhou et al. found indicated that CircRNA-ENO1 could promote glycolysis and tumor progression in LUAD through upregulating its host gene *ENO1* [23]. The difference in prognosis between high-risk and low-risk patients may be the result of the interaction of these pathways.

Underlying genomic burden lead to the diverse performance of patients. Therefore, we found that the *MAS1L* was negatively correlated with the TMB and mRNAsi. In brief, TMB is the number of mutations in tumors, which can reflect the instability of the genome to some extent [24]. Generally, a higher TMB level in the tumor microenvironment can increase the intratumoral heterogeneity, making cancer cells more aggressive [25]. Tumor stemness index, like mRNAsi, is an index to evaluate the similarity between tumor cells and stem cells, which is associated with the active biological processes in stem cells and a higher degree of tumor dedifferentiation [26]. In lung cancer, Hong et al. found that the circular RNA circ_CPA4 could promote lung cancer proliferation, stemness, drug resistance, and immune evasion through the miR-let-7/PD-L1 axis [27]. Interestingly, Schaal et al. revealed that nicotine and electronic cigarettes could promote self-renewal of stem-like side-population cells, implicated in the dormancy, metastasis, and drug resistance in lung cancer [28].

Recently, the microenvironment of tumor cells located in has gained increasing attention from researchers. Immune cells are an important component of tumor microenvironment. Our result showed that *MAS1L* was positively correlated with the mast cells, eosinophils, iDC, DC, and M1 macrophages, while negatively correlated with Th2 cells. Eosinophils are rare multifunctional granulocytes and have been reported to play an antitumor role in cancer. Through manipulating eosinophil-related cytokines *CCL11* and *IL-5*, Simson et al. found a negative correlation between tumor growth and eosinophil infiltration [29]. The activation of activated eosinophils promotes tumor rejection through recruitment, activation, and maturation of several immune cells in addition to its direct cytotoxic actions on cancer cells [30]. Carretero et al. indicated that eosinophils could recruit cytotoxic CD8+ T cells to promote tumor rejection [31].

Some limitations should be noticed. Firstly, the patients enrolled in our study were predominantly western populations, which might lead to underlying race bias and reduce the credibility of our conclusions. Secondly, the location of lymph node metastasis is not fully provided. If the relevant data is further improved in the future, this will increase the stability of our conclusions.

## Figures and Tables

**Table 1:** Baseline information of enrolled patients.

Features		Numbers	Percentage (%)
Age	<= 65	241	46.2%
	> 65	262	50.2%
	Unknown	19	3.6%

Gender	Female	280	53.6%
	Male	242	46.4%

Stage	Stage I	279	53.4%
	Stage II	124	23.8%
	Stage III	85	16.3%
	Stage IV	26	5.0%
	Unknown	8	1.5%

T-stage	T1	172	33.0%
	T2	281	53.8%
	T3	47	9.0%
	T4	19	3.6%
	Unknown	3	0.6%

M-stage	M0	353	67.6%
	M1	25	4.8%
	Unknown	144	27.6%

N-stage	N0	335	64.2%
	N1	98	18.8%
	N2	75	14.4%
	N3	2	0.4%
	Unknown	12	2.3%

## Data Availability

The datasets used and/or analysed during the current study are available from the corresponding author on reasonable request.

## Supplementary Materials

Supplementary Materials Figure S1: immunotherapy difference between high- and low-risk patients. Notes: (a) correlation between the riskscore and TIDE; (b) TIDE difference between high- and low-risk patients; (c–d) the percentage of immunotherapy responders and nonresponders in high- and low-risk patients. Figure S2: biological enrichment of high- and low-risk patients. Notes: (a) GO analysis; (b) KEGG analysis. Figure S3: biological enrichment of patients with high and low MAS1L expression. Notes: (a) GO analysis; (b) KEGG analysis.
