# Supplementary material for: Identification of the Characteristic Genes and their Roles in Lung Adenocarcinoma Lymph Node Metastasis through Machine Learning Algorithm
Source: Comput Math Methods Med. 2022 Oct 12;2022:1968829. doi: 10.1155/2022/1968829 (PMC9581663; doi:10.1155/2022/1968829)

# Figure S1. Immunotherapy difference between high and low risk patients

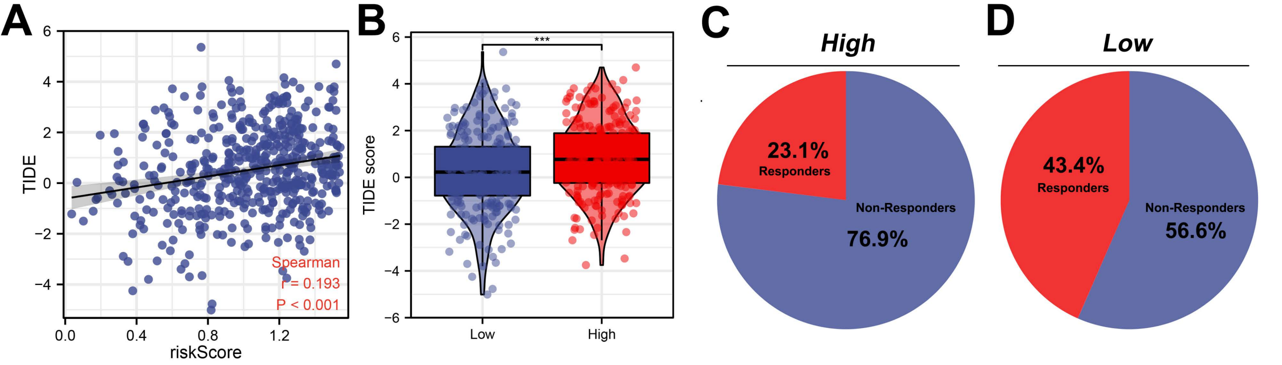

# Figure S2. Biological enrichment of high and low risk patients

**A**

| Pathway | NES | pval | padj |
|---|---|---|---|
| GOBP_DNA_REPLICATION_CHECKPOINT_SIGNALING | 2.47 | 7.7e−04 | 9.7e−02 |
| GOBP_DNA_STRAND_ELONGATION_INVOLVED_IN_DNA_REPLICATION | 2.35 | 7.7e−04 | 9.7e−02 |
| GOBP_POSITIVE_REGULATION_OF_TELOMERASE_RNA_LOCALIZATION_TO_CAJAL_BODY | 2.25 | 7.7e−04 | 9.7e−02 |
| GOMF_DNA_REPLICATION_ORIGIN_BINDING | 2.33 | 7.7e−04 | 9.7e−02 |
| GOBP_ANAPHASE_PROMOTING_COMPLEX_DEPENDENT_CATABOLIC_PROCESS | 2.04 | 8.0e−04 | 9.7e−02 |
| GOBP_ESTABLISHMENT_OF_PROTEIN_LOCALIZATION_TO_TELOMERE | 2.30 | 8.4e−04 | 9.7e−02 |
| GOBP_KINETOCHORE_ASSEMBLY | 2.42 | 8.4e−04 | 9.7e−02 |
| GOBP_MITOTIC_CHROMOSOME_CONDENSATION | 1.97 | 8.4e−04 | 9.7e−02 |
| GOCC_PROTEASOME_CORE_COMPLEX | 2.39 | 8.4e−04 | 9.7e−02 |
| GOBP_MITOCHONDRIAL_CYTOCHROME_C_OXIDASE_ASSEMBLY | 2.06 | 9.1e−04 | 9.7e−02 |
| GOCC_INTRINSIC_COMPONENT_OF_SYNAPTIC_MEMBRANE | −1.48 | 4.0e−04 | 9.7e−02 |
| GOMF_G_PROTEIN_COUPLED_RECEPTOR_ACTIVITY | −1.31 | 4.0e−04 | 9.7e−02 |
| GOBP_REGULATION_OF_MEMBRANE_POTENTIAL | −1.32 | 4.0e−04 | 9.7e−02 |
| GOMF_CATION_CHANNEL_ACTIVITY | −1.37 | 3.0e−04 | 9.7e−02 |
| GOCC_TRANSPORTER_COMPLEX | −1.33 | 3.0e−04 | 9.7e−02 |
| GOMF_ACETYLCHOLINE_RECEPTOR_ACTIVITY | −1.75 | 2.2e−04 | 9.7e−02 |
| GOCC_CATION_CHANNEL_COMPLEX | −1.44 | 2.0e−04 | 9.7e−02 |
| GOCC_INTRINSIC_COMPONENT_OF_PRESYNAPTIC_MEMBRANE | −1.72 | 1.0e−04 | 9.7e−02 |
| GOBP_EXPORT_ACROSS_PLASMA_MEMBRANE | −1.61 | 1.0e−04 | 9.7e−02 |
| GOCC_T_CELL_RECEPTOR_COMPLEX | −1.65 | 1.0e−04 | 9.7e−02 |

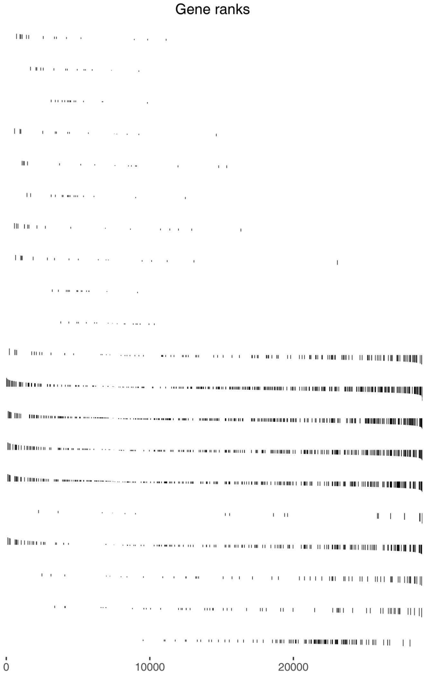


**B**

| Pathway | NES | pval | padj |
|---|---|---|---|
| KEGG_MISMATCH_REPAIR | 2.17 | 1.1e−03 | 5.5e−02 |
| KEGG_CITRATE_CYCLE_TCA_CYCLE | 2.20 | 1.3e−03 | 5.5e−02 |
| KEGG_HOMOLOGOUS_RECOMBINATION | 2.37 | 1.3e−03 | 5.5e−02 |
| KEGG_DNA_REPLICATION | 2.78 | 1.7e−03 | 5.9e−02 |
| KEGG_PROTEASOME | 2.89 | 2.1e−03 | 6.0e−02 |
| KEGG_RIBOSOME | 2.18 | 5.6e−03 | 1.4e−01 |
| KEGG_P53_SIGNALING_PATHWAY | 1.65 | 9.2e−03 | 1.7e−01 |
| KEGG_NUCLEOTIDE_EXCISION_REPAIR | 1.64 | 1.3e−02 | 1.8e−01 |
| KEGG_BASE_EXCISION_REPAIR | 1.51 | 1.4e−02 | 1.8e−01 |
| KEGG_PROTEIN_EXPORT | 1.64 | 1.5e−02 | 1.8e−01 |
| KEGG_CARDIAC_MUSCLE_CONTRACTION | −1.28 | 7.1e−02 | 4.5e−01 |
| KEGG_DRUG_METABOLISM_CYTOCHROME_P450 | −1.29 | 6.6e−02 | 4.3e−01 |
| KEGG_VASCULAR_SMOOTH_MUSCLE_CONTRACTION | −1.24 | 6.0e−02 | 4.1e−01 |
| KEGG_RENIN_ANGIOTENSIN_SYSTEM | −1.37 | 6.0e−02 | 4.1e−01 |
| KEGG_DILATED_CARDIOMYOPATHY | −1.27 | 5.8e−02 | 4.1e−01 |
| KEGG_ALPHA_LINOLENIC_ACID_METABOLISM | −1.38 | 5.5e−02 | 4.1e−01 |
| KEGG_LINOLEIC_ACID_METABOLISM | −1.48 | 1.5e−02 | 1.8e−01 |
| KEGG_PROXIMAL_TUBULE_BICARBONATE_RECLAMATION | −1.50 | 1.4e−02 | 1.8e−01 |
| KEGG_FATTY_ACID_METABOLISM | −1.50 | 7.1e−03 | 1.5e−01 |
| KEGG_NEUROACTIVE_LIGAND_RECEPTOR_INTERACTION | −1.39 | 5.0e−04 | 5.5e−02 |

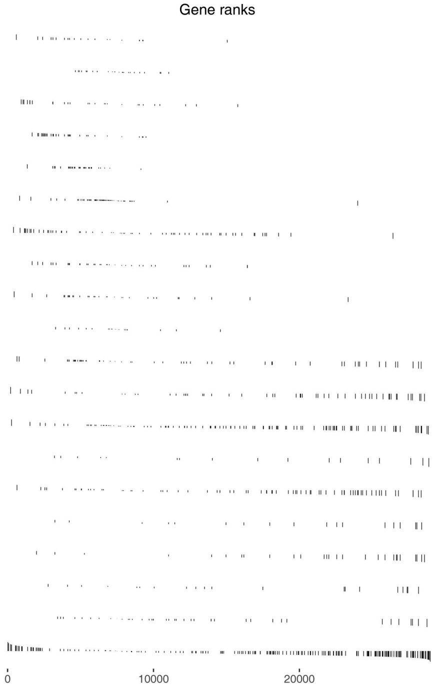

# Figure S3. Biological enrichment of patients with high and low MAS1L expression

## A

| Pathway | NES | pval | padj |
|---|---|---|---|
| GOMF_RNA_BINDING_INVOLVED_IN_POSTTRANSCRIPTIONAL_GENE_SILENCING | 1.66 | 1.0e−04 | 3.0e−02 |
| GOCC_T_CELL_RECEPTOR_COMPLEX | 1.97 | 1.0e−04 | 3.0e−02 |
| GOCC_PLASMA_MEMBRANE_SIGNALING_RECEPTOR_COMPLEX | 1.42 | 4.0e−04 | 3.0e−02 |
| GOMF_BITTER_TASTE_RECEPTOR_ACTIVITY | 1.68 | 1.1e−03 | 3.0e−02 |
| GOBP_SPLICEOSOMAL_TRI_SNRNP_COMPLEX_ASSEMBLY | 1.66 | 2.0e−03 | 3.1e−02 |
| GOCC_CAJAL_BODY | 1.52 | 3.1e−03 | 3.3e−02 |
| GOCC_MHC_CLASS_II_PROTEIN_COMPLEX | 1.62 | 3.3e−03 | 3.5e−02 |
| GOBP_B_CELL_RECEPTOR_SIGNALING_PATHWAY | 1.44 | 5.0e−03 | 4.2e−02 |
| GOMF_PRE_MRNA_5_SPLICE_SITE_BINDING | 1.58 | 6.5e−03 | 4.7e−02 |
| GOBP_POSITIVE_REGULATION_OF_CELL_KILLING | 1.50 | 6.9e−03 | 4.8e−02 |
| GOMF_D_THREO_ALDOSE_1_DEHYDROGENASE_ACTIVITY | −2.06 | 4.5e−04 | 3.0e−02 |
| GOMF_PROTON_TRANSPORTING_ATP_SYNTHASE_ACTIVITY_ROTATIONAL_MECHANISM | −2.02 | 4.4e−04 | 3.0e−02 |
| GOBP_ESTABLISHMENT_OF_PROTEIN_LOCALIZATION_TO_TELOMERE | −2.17 | 4.4e−04 | 3.0e−02 |
| GOBP_DNA_REPLICATION_CHECKPOINT_SIGNALING | −2.05 | 4.4e−04 | 3.0e−02 |
| GOBP_TRNA_5_END_PROCESSING | −2.10 | 4.2e−04 | 3.0e−02 |
| GOBP_SEQUESTERING_OF_METAL_ION | −2.12 | 4.2e−04 | 3.0e−02 |
| GOBP_PROGESTERONE_METABOLIC_PROCESS | −2.07 | 4.2e−04 | 3.0e−02 |
| GOBP_POSITIVE_REGULATION_OF_TELOMERASE_RNA_LOCALIZATION_TO_CAJAL_BODY | −2.19 | 4.1e−04 | 3.0e−02 |
| GOBP_POSITIVE_REGULATION_OF_MEIOTIC_NUCLEAR_DIVISION | −2.09 | 4.1e−04 | 3.0e−02 |
| GOBP_DNA_STRAND_ELONGATION_INVOLVED_IN_DNA_REPLICATION | −2.05 | 4.1e−04 | 3.0e−02 |

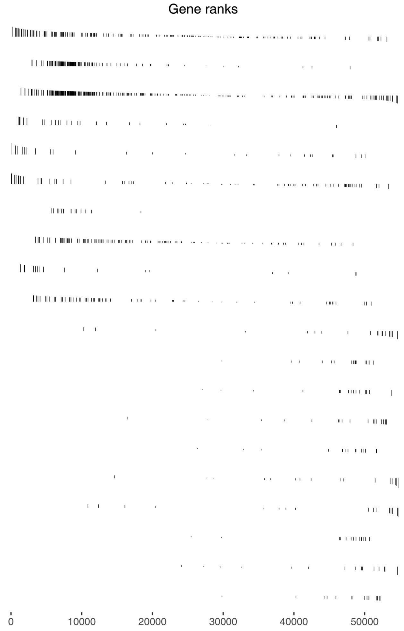


## B

| Pathway | NES | pval | padj |
|---|---|---|---|
| KEGG_INTESTINAL_IMMUNE_NETWORK_FOR_IGA_PRODUCTION | 1.68 | 4.5e−04 | 1.3e−02 |
| KEGG_ASTHMA | 1.69 | 7.2e−04 | 1.3e−02 |
| KEGG_ALLOGRAFT_REJECTION | 1.60 | 2.7e−03 | 2.0e−02 |
| KEGG_HEMATOPOIETIC_CELL_LINEAGE | 1.44 | 1.0e−02 | 3.8e−02 |
| KEGG_VIRAL_MYOCARDITIS | 1.41 | 2.3e−02 | 7.2e−02 |
| KEGG_AUTOIMMUNE_THYROID_DISEASE | 1.33 | 7.4e−02 | 1.9e−01 |
| KEGG_RENIN_ANGIOTENSIN_SYSTEM | 1.36 | 8.8e−02 | 2.1e−01 |
| KEGG_PRIMARY_IMMUNODEFICIENCY | 1.32 | 9.7e−02 | 2.3e−01 |
| KEGG_GRAFT_VERSUS_HOST_DISEASE | 1.31 | 9.8e−02 | 2.3e−01 |
| KEGG_CELL_ADHESION_MOLECULES_CAMS | 1.22 | 1.0e−01 | 2.3e−01 |
| KEGG_STARCH_AND_SUCROSE_METABOLISM | −1.97 | 1.0e−03 | 1.3e−02 |
| KEGG_DRUG_METABOLISM_OTHER_ENZYMES | −1.88 | 1.0e−03 | 1.3e−02 |
| KEGG_PROTEASOME | −2.46 | 9.4e−04 | 1.3e−02 |
| KEGG_PORPHYRIN_AND_CHLOROPHYLL_METABOLISM | −2.24 | 8.4e−04 | 1.3e−02 |
| KEGG_AMINOACYL_TRNA_BIOSYNTHESIS | −2.16 | 8.4e−04 | 1.3e−02 |
| KEGG_DNA_REPLICATION | −2.31 | 7.4e−04 | 1.3e−02 |
| KEGG_BASE_EXCISION_REPAIR | −1.99 | 7.4e−04 | 1.3e−02 |
| KEGG_CITRATE_CYCLE_TCA_CYCLE | −2.01 | 6.6e−04 | 1.3e−02 |
| KEGG_PENTOSE_AND_GLUCURONATE_INTERCONVERSIONS | −2.27 | 6.0e−04 | 1.3e−02 |
| KEGG_ASCORBATE_AND_ALDARATE_METABOLISM | −2.33 | 5.5e−04 | 1.3e−02 |

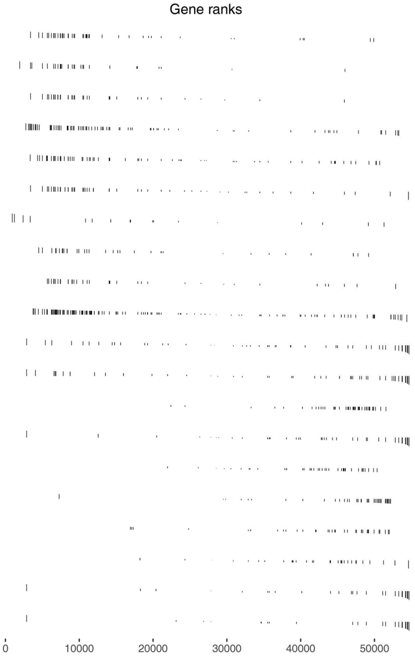

Supplement: Supplementary Materials — Figure S1: immunotherapy difference between high- and low-risk patients. Notes: (a) correlation between the riskscore and TIDE; (b) TIDE difference between high- and low-risk patients; (c–d) the percentage of immunotherapy responders and nonresponders in high- and low-risk patients. Figure S2: biological enrichment of high- and low-risk patients. Notes: (a) GO analysis; (b) KEGG analysis. Figure S3: biological enrichment of patients with high and low MAS1L expression. Notes: (a) GO analysis; (b) KEGG analysis. [file 1968829.f1.pdf]
